# SARS Vaccine Development

**DOI:** 10.3201/eid1107.050219

**Published:** 2005-07

**Authors:** Shibo Jiang, Yuxian He, Shuwen Liu

**Affiliations:** * New York Blood Center, New York, New York, USA

**Keywords:** SARS, SARS-CoV, spike protein, receptor-binding domain, Neutralizing epitone, vaccine

## Abstract

Developing effective and safe vaccines is urgently needed to prevent infection by severe acute respiratory syndrome (SARS)–associated coronavirus (SARS-CoV). The inactivated SARS-CoV vaccine may be the first one available for clinical use because it is easy to generate; however, safety is the main concern. The spike (S) protein of SARS-CoV is the major inducer of neutralizing antibodies, and the receptor-binding domain (RBD) in the S1 subunit of S protein contains multiple conformational neutralizing epitopes. This suggests that recombinant proteins containing RBD and vectors encoding the RBD sequence can be used to develop safe and effective SARS vaccines.

Severe acute respiratory syndrome (SARS) is a newly emerged infectious disease caused by SARS-associated coronavirus (SARS-CoV) (1). It originated in the Guangdong province of China in late 2002, spread rapidly around the world along international air-travel routes, and resulted in 8,450 cases and 810 deaths in 33 countries and areas on 5 continents (http://www.cdc.gov/mmwr/mguide_sars.html). The global outbreak of SARS seriously threatened public health and socioeconomic stability worldwide. Although this outbreak was eventually brought under control in 2003, several isolated outbreaks of SARS subsequently occurred because of accidental releases of the SARS-CoV isolates from laboratories in Taiwan, Singapore, and mainland China (http://www.who.int/csr/sars/en/). In late 2003 and early 2004, new infections in persons who had contact with animals infected with SARS-CoV strains significantly different from those predominating in the 2002–2003 outbreak were reported in Guangdong, China (1). These events indicate that a SARS epidemic may recur at any time in the future, either by the virus escaping from laboratory samples or by SARS-CoV isolates evolving from SARS-CoV–like virus in animal hosts.

## Origin and Evolution of SARS-CoV

Coronaviruses of the genus *Coronavirus* can be divided into 3 antigenic groups. Group 1 consists of human coronavirus 229E (HCoV-229E), porcine epidemic diarrhea virus, and feline infectious peritonitis virus (FIPV). Group 2 includes bovine coronavirus, murine hepatitis virus, and human coronavirus OC34 (HCoV-OC43). Group 3 contains avian infectious bronchitis virus. SARS-CoV is a new member of the genus *Coronavirus*, but it does not belong to any of the 3 antigenic groups, although some reports suggest that it most resembles the group 2 coronavirus (2). SARS-CoV may have originated in animals. SARS-CoV–like virus with >99% nucleotide homology with human SARS-CoV was identified in palm civets and other animals found in live animal markets in Guangdong, China (3). The SARS-CoV–like virus that exists in animals does not cause typical SARS-like disease in the natural hosts and is not transmitted from animals to humans. Under certain conditions, the virus may have evolved into the early human SARS-CoV, with the ability to be transmitted from animals to humans or even from humans to humans, resulting in localized outbreaks and mild human disease. Under selective pressure in humans, the early human SARS-CoV may further evolve into the late human SARS-CoV, which can cause local or even global outbreaks and typical SARS in humans with high death rates. Early human SARS-CoV is closer genetically to animal SARS-CoV–like virus than to late human SARS-CoV, which has a 29-nucleotide (in some isolates a 415-nucleotide) deletion in open reading frame 8 (3,4). The characteristics of these viruses are summarized in the Table (4–6).

**Table:** Comparison of civet SARS-CoV-like virus and the early and late human SARS-CoV*

Characteristics	SARS-CoV–like virus	Early human SARS-CoV	Late human SARS-CoV
Transmission	Animal-to-animal	Animal/human-to-human	Human-to-human
Outbreak	No	No/local	Local/global
Causes disease	No	Mild	Severe
Representative strains	SZ3, SZ16	GD03T0013	Tor2, Urbani, BJ01, GZ02
Source	Palm civets	SARS patients during 2003–2004 epidemic	SARS patients during 2002–2003 outbreak
29-nucleotide deletion	No	No	Yes (some have a 415– nucleotide deletion)
Properties of spike protein			
Genetic homogenicity	Low	Low	High
Rate of nonsynonymous mutation	High	High	Low
Binding affinity to ACE2	Low	Low	High

*Information was obtained from references 4–6. SARS-COV, severe acute respiratory syndrome–associated coronavirus; ACE2, angiotensin-converting enzyme 2.

**Figure 1:**
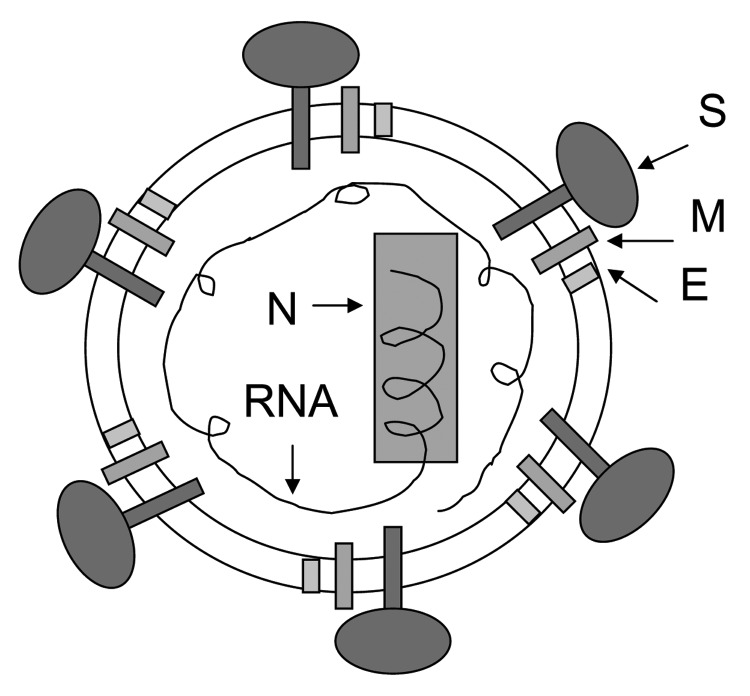
Strategy for designing vaccines for severe acute respiratory syndrome (SARS) using inactivated SARS-associated coronavirus. This virus expresses several structural proteins, including nucleocapsid (N), membrane (M), envelope (E), and spike (S).

**Figure 2:**
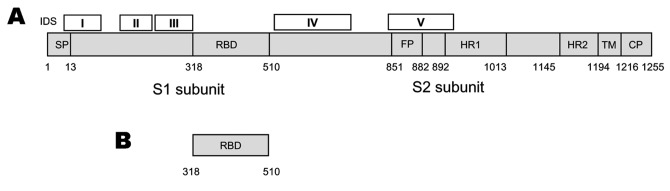
Strategies for designing vaccines for severe acute respiratory syndrome (SARS) using A) spike (S) protein and B) fragments containing neutralizing epitopes. SP, signal peptide; RBD, receptor binding domain; FP, fusion peptide; HR, heptad repeat; TM, transmembrane domain; CP, cytoplasm domain. IDS, immunodominant sites I to V corresponding to the sequences of amino acid residues 9–71, 171–224, 271–318, 528–635, and 842–913, respectively. The residue numbers of each region correspond to their positions in the S protein of SARS–associated coronavirus (SARS-CoV) strain Tor2. RBD contains the major neutralizing epitopes in the S protein. The recombinant RBD may be used as an efficacious and safe vaccine for preventing infection by SARS-CoV strains with distinct genotypes.

SARS-CoV can be efficiently grown in cell culture (1) and rapidly spread from person to person (7). It can survive in feces and urine at room temperature for >2 days (http://www.who.int/csr/sars/en) and may cause serious, even fatal, disease. SARS-CoV, a National Institute of Allergy and Infectious Diseases Biodefense Category C priority pathogen (http://www2.niaid.nih.gov/Biodefense/bandc_priority.htm) could be used by bioterrorists as a biological weapon. Therefore, development of effective and safe vaccines is urgently needed to prevent a new SARS epidemic and for biodefense preparedness. Currently, 3 major classes of SARS vaccines are under development: 1) inactivated SARS-CoV (Figure 1), 2) full-length S protein (Figure 2A), and 3) those based on fragments containing neutralizing epitopes (Figure 2B).

### Inactivated SARS-CoV–based Vaccines

SARS-CoV expresses several structural proteins, including nucleocapsid, membrane, envelope, and spike (S) proteins (1). All may serve as antigens to induce neutralizing antibodies and protective responses. In general, prior to identification of the protein that contains the major neutralizing epitopes, the inactivated virus may be used as the first-generation vaccine because it is easy to generate whole killed virus particles. However, once the neutralizing epitopes are identified, the inactivated virus vaccine should be replaced by vaccines based on fragments containing neutralizing epitopes since they are safer and more effective. Several reports have showed that SARS-CoV inactivated with formaldehyde, UV light, and β-propiolactone can induce virus-neutralizing antibodies in immunized animals (8–11), and the first inactivated SARS-CoV vaccine is being tested in the clinical trials in China. However, safety of the inactivated vaccine is a serious concern; production workers are at risk for infection during handling of concentrated live SARS-CoV, incomplete virus inactivation may cause SARS outbreaks among the vaccinated populations, and some viral proteins may induce harmful immune or inflammatory responses, even causing SARS-like diseases (12,13).

### S Protein–based Vaccines

The S protein of SARS-CoV, a type I transmembrane glycoprotein, is responsible for virus binding, fusion, and entry and is a major inducer of neutralizing antibodies (1,14). S protein consists of a signal peptide (SP: amino acids [aa] 1–12) and 3 domains: an extracellular domain (aa 13–1193), a transmembrane domain (aa 11194–1215), and an intracellular domain (aa 1216–1255). Its extracellular domain consists of 2 subunits, S1 and S2 (14), although the cleavage site between these subunits has not been clearly defined. The S1 subunit is responsible for virus binding to the receptor, angiotensin-converting enzyme 2 (ACE2) (15,16). A fragment located in the middle region of the S1 subunit (aa 318–510) is the receptor-binding domain (RBD) for ACE2 (17–19). SARS-CoV may also bind to cells through the alternative receptors DC-SIGN or L-SIGN (20,21), but the binding sites for these alternative receptors have not been defined. The S2 subunit, which contains a putative fusion peptide and 2 heptad repeats (HR1 and HR2), is responsible for fusion between the viral and target cell membranes. Infection by SARS-CoV is initiated by binding of RBD in the viral S protein S1 subunit to ACE2 on target cells. This forms a fusogenic core between the HR1 and HR2 regions in the S2 domain that brings the viral and target cell membranes into close proximity, which results in virus fusion and entry (22–24). This scenario indicates that the S protein may be used as a vaccine to induce antibodies for blocking virus binding and fusion.

Several recombinant vector-based vaccines expressing SARS-CoV S protein have been assessed in preclinical studies. Yang et al. (25) reported that a candidate DNA vaccine encoding the full-length S protein induced neutralizing antibodies (neutralizing titers ranging from 1:50 to 1:150) and protected mice from SARS-CoV challenge. Using DNA vaccines encoding the full-length and segments of S protein to immunize rabbits, Wang et al. have produced higher titers of neutralizing antibodies and demonstrated that major and minor neutralizing epitopes are located in the S1 and S2 subunits, respectively (26). Other groups also found neutralizing epitopes in the S2 subunit (27,28). Bisht et al. (29) have shown that intranasal or intramuscular inoculations of mice with highly attenuated modified vaccinia virus Ankara (MVA) vaccines encoding full-length SARS-CoV S protein also produce neutralizing antibodies with mean neutralizing titers of 1:284. Bukreyev et al. (30) reported that mucosal immunization of African green monkeys with an attenuated parainfluenza virus expressing S protein resulted in production of neutralizing antibodies and protected animals from infection by challenge with SARS-CoV. These data suggest that the S protein can induce neutralizing antibodies and protective responses in immunized animals.

Using convalescent-phase sera from SARS patients and a set of peptides spanning the entire sequence of the SARS-CoV S protein, we have identified 5 linear immunodominant sites (IDS) in the S protein (Figure 2A). IDS I, II, III, and V reacted with >50% of the convalescent-phase sera from SARS patients, while IDS IV was reactive with >80% of SARS sera, suggesting that IDS IV is the major immunodominant epitope on the S protein (31). Synthetic peptides corresponding to IDS could induce high titers of S protein–specific antibodies, but none of these antibodies possesses neutralizing activity. These findings suggest that the IDS in S protein may not induce neutralizing antibodies. Whether these antibodies enhance infection by heterologous SARS-CoV strains or mediate harmful immune responses is unclear. The S protein of FIPV expressed by recombinant vaccinia can cause antibody-dependent enhancement of disease if vaccinated animals are subsequently infected with wild-type virus (32). Our previous studies on HIV-1 showed that antibodies against some immunodominant epitopes in the HIV-1 envelope glycoprotein could enhance infection by heterologous HIV-1 strains (33). Most recently, Yang et al. (6) demonstrated that the polyclonal and monoclonal antibodies against S protein of the late SARS-CoV (Urbani strain) could neutralize infection by the relevant late SARS-CoV strains. However, these antibodies enhanced infection by an early human SARS-CoV isolate (GD03T0013) and the civet SARS-CoV–like viruses. These investigators have shown that the ACE2-binding domain mediates the antibody-dependent enhancement of civet SARS-CoV–like virus entry (6). Theoretically, some antibodies to the ACE2-binding domain may enhance infection if these antibodies closely mimic the receptor ACE2 and induce similar conformational changes, as the receptor likely does. The S protein with truncation at aa 1153 failed to cause antibody-dependent enhancement of infection, although it still induced neutralizing antibodies. This finding suggests that removal of the aa 1153–1194 region may abrogate induction of virus infection–enhancing antibodies (6). Vaccination of ferrets with MVA-based SARS vaccine expressing full-length S protein caused liver damage after animals were challenged with SARS-CoV (34). These findings raised concerns about the efficacy and safety of the vaccines containing or expressing full-length S protein.

### Vaccines Based on Fragments Containing Neutralizing Epitopes

RBD, a fragment (≈193 aa residues) in the middle of S1 subunit of S protein (Figure 2B), is responsible for virus binding to the receptor on target cells. We have demonstrated that the antisera from SARS patients and from animals immunized with inactivated SARS-CoV reacted strongly with RBD (9,35). Absorption of antibodies by RBD from these antisera results in the removal of most of the neutralizing antibodies, and RBD-specific antibodies isolated from these antisera have potent neutralizing activity (35,36). We have also shown that rabbits and mice immunized with RBD produced high titers of neutralizing antibodies against SARS-CoV with 50% neutralizing titers at a >1:10,000 serum dilution (37). The immunized mice were protected from SARS-CoV challenge (unpub. data). The antibodies purified from the antisera against SARS-CoV significantly inhibited RBD binding to ACE2 (9,36–38). Using spleen cells from mice immunized with RBD, we have generated a panel of 25 monoclonal antibodies (MAbs) that recognize different conformational epitopes on RBD and possess potent neutralizing activity (38). Our result is in agreement with the report by van den Brink et al. (39), who identified 3 human neutralizing anti-S MAbs from antibody phage display libraries by using inactivated SARS-CoV as the target. These researchers also found that all of these MAbs specifically bound to RBD and blocked interaction between RBD and ACE2. These findings suggest that RBD contains the major neutralizing epitopes in the S protein and is an ideal SARS vaccine candidate because RBD contains the receptor-binding site, which is critical for virus attachment to the target cell for infection (15,17–19). Antibodies specific for RBD are expected to block binding of virus to the target cell. RBD induces higher titers of neutralizing antibodies than those vaccines expressing the full-length S protein (25,26,29,30,37,38). RBD sequences among the late SARS-CoV strains are highly conserved. When the early and late SARS-CoV strains are compared, only 3 to 5 aa residues are variable among the 193 residues in RBD and most of the isolates vary by only 1 residue (4). van den Brink et al. (39) showed that 1 human MAb (CR3014) specific for RBD of SARS-CoV strain FM1 can effectively bind to most RBDs of the early and late SARS-CoV strains. These data suggest that antibodies directed against RBD of a SARS-CoV isolate may neutralize infection by a broad spectrum of SARS-CoV strains. Therefore, recombinant proteins containing RBD or vectors encoding RBD may be used as vaccines for preventing infection by SARS-CoV with distinct genotypes.

## Conclusions

An ideal SARS vaccine should 1) elicit highly potent neutralizing antibody responses against a broad spectrum of viral strains; 2) induce protection against infection and transmission; and 3) be safe by not inducing any infection-enhancing antibodies or harmful immune or inflammatory responses. Currently, an inactivated SARS-CoV vaccine is in clinical trials in China. Safety is the major concern for this type of vaccine (12). The S protein is the major inducer of neutralizing antibodies. Recombinant vector-based vaccines expressing full-length S protein of the late SARS-CoV are under development. These vaccines can induce potent neutralizing and protective responses in immunized animals but may induce antibodies that enhance infection by early human SARS-CoV and animal SARS-CoV–like viruses (6). Recent studies have demonstrated that recombinant RBD consists of multiple conformational neutralizing epitopes that induce highly potent neutralizing antibodies against SARS-CoV (9,26,35–38). Unlike full-length S protein, RBD does not contain immunodominant sites that induce nonneutralizing antibodies. RBD sequences are relatively conserved. Thus, recombinant RBD or vectors encoding RBD may be used as safe and efficacious vaccines for preventing infection by SARS-CoV with distinct genotypes.

Dr. Jiang is associate member and head of the Viral Immunology Laboratory, Lindsley F. Kimball Research Institute, New York Blood Center. His primary research interests include development of vaccines and therapeutic agents against SARS-CoV and HIV.
